# High S100A9^+^ cell density predicts a poor prognosis in hepatocellular carcinoma patients after curative resection

**DOI:** 10.18632/aging.203162

**Published:** 2021-06-22

**Authors:** Jing Liao, Jin-Zhu Li, Jing Xu, Yongquan Xu, Wei-Ping Wen, Limin Zheng, Lian Li

**Affiliations:** 1 MOE Key Laboratory of Gene Function and Regulation, School of Life Sciences, Sun Yat-sen University, Guangzhou, Guangdong 510275, China; 2 Division of Head and Neck Surgery, Department of Otorhinolaryngology, The Sixth Affiliated Hospital, Sun Yat-sen University, Guangzhou, Guangdong 510655, China; 3 Guangdong Institute of Gastroenterology, The Sixth Affiliated Hospital, Sun Yat-sen University, Guangzhou, Guangdong 510655, China; 4 State Key Laboratory of Oncology in South China, Collaborative Innovation Center for Cancer Medicine, Sun Yat-sen University Cancer Center, Guangzhou, Guangdong 510060, China

**Keywords:** hepatocellular carcinoma, S100A9, myeloid cells, prognosis, curative resection

## Abstract

S100A9 is differentially expressed in various cell types and is associated with the development, progression and metastasis of various cancers. However, the expression, distribution, and clinical significance of S100A9 in hepatocellular carcinoma (HCC) remain unclear. In the present study, The Cancer Genome Atlas (TCGA) database was used to examine *S100A9* gene expression in HCC; we found that *S100A9* expression was associated with HCC prognosis. In addition, S100A9 protein expression was assessed by immunohistochemistry analysis of tissues from 382 HCC patients. We found that the infiltration of S100A9^+^ cells in both tumor and nontumor tissues could predict poor overall survival (*P* = 0.0329, tumor; *P* = 0.0003, nontumor) and a high recurrence risk (*P* = 0.0387, tumor; *P* = 0.0015, nontumor) in our tissue microarray analysis. Furthermore, immunofluorescence double staining revealed that the primary S100A9-expressing cells in adjacent nontumoral tissue were CD15^+^ neutrophils, and both CD68^+^ macrophages and CD15^+^ neutrophils expressed S100A9 in HCC tumor tissues. Taken together, the results suggest that high S100A9^+^ cell density predicts a poor prognosis in HCC patients, and S100A9 expression could potentially serve as an independent prognostic marker for HCC.

## INTRODUCTION

Hepatocellular carcinoma (HCC) is one of the leading causes of cancer-related death worldwide [1, 2]. Currently, surgical resection, orthotopic liver transplantation, immunotherapy and radiofrequency thermal ablation are the most common treatments for HCC [3]. Despite significant advances, the incidence and mortality rates of HCC continue to rise. Hence, there is an urgent need for a reliable prognostic biomarker to effectively stratify patients for appropriate treatment and to improve patient survival [4–6].

Accumulating evidence has highlighted the significance of inflammation, which dramatically affects the progression and prognosis of HCC patients. S100A9 is a calcium-binding protein that plays an indispensable role as a mediator in inflammatory processes [7]. Evidence has demonstrated that S100A9 is elevated in various solid tumors and that the upregulation of S100A9 positively correlates with poor outcomes in colorectal, gastric, liver, pancreatic and prostate cancer [8–14]. Moreover, some researchers have observed that S100A9 is a negative regulator of lymph node metastasis in gastric adenocarcinoma [15, 16]. These findings suggest that S100A9 exerts antitumor or tumorigenic activity depending on the cancer type and could serve as a potential biomarker for prognosis prediction in cancer.

Solid tumors comprise not only cancer cells but also many other nonmalignant cell types, which produce a unique microenvironment that regulates tumor progression. S100A9 is expressed mainly by neutrophils, monocytes, and activated macrophages [7, 8]. Recently, S100A9 was also proposed as a novel marker of human monocytic myeloid-derived suppressor cells (MDSCs) [17]. Thus, it is important to define the cellular sources of S100A9 in HCC and evaluate its association with clinicopathological factors. Herein, we investigated the expression, distribution and prognostic significance of S100A9 in 382 patients with HCC. Our data showed that most S100A9-expressing cells were neutrophils and macrophages (Mφs) in tumor tissues, and neutrophils in nontumoral tissues. Moreover, we found that either tumoral or nontumoral S100A9^+^ cell density can serve as an independent predictor of poor prognosis in patients with HCC.

## RESULTS

### High S100A9 expression correlates with a poor prognosis in HCC patients in the TCGA-LIHC dataset

**Figure 1:**
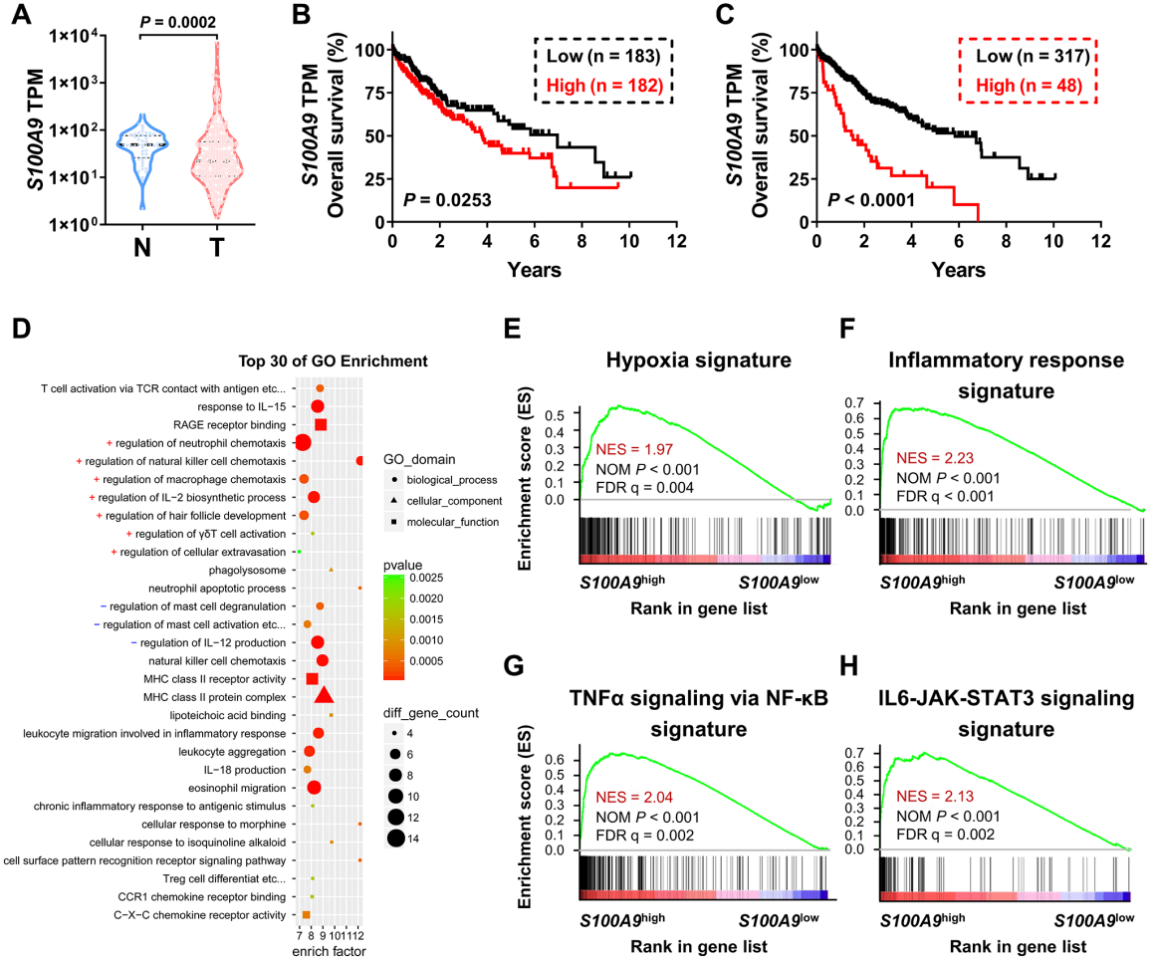
**Association between *S100A9* expression and prognosis of HCC patients in the TCGA-LIHC database.** (**A**) Violin plot showing the differential expression of the *S100A9* gene in tumor (*n* = 365) or nontumor (*n* = 50) tissues from the TCGA-LIHC database; the Mann- Whitney test was used to analyze the nonparametric test between the two groups. HCC patients in the TCGA dataset were divided into two groups according to the median value of *S100A9* expression (**B**) or minimum *P*-value approach (**C**). The prognostic value of *S100A9* was evaluated by the Kaplan-Meier method, and examined by the log-rank test. (**D**) Top 30 GO enrichment results of the 3493 genes highly expressed in the *S100A9*^high^ group. (**E**–**H**) Top enriched signaling pathways based on *S100A9* expression identified by GSEA.

We used bioinformatics analysis to examine the association between S100A9 expression and the prognosis of patients with HCC. According to the TCGA-LIHC dataset analysis, *S100A9* gene expression was significantly downregulated in tumor tissues compared to adjacent nontumor tissues (Figure 1A). Moreover, high S100A9 expression in tumor tissue correlated with an unfavorable prognosis in HCC patients in subgroups divided by the median value (Figure 1B) and minimum *P*-value approach (Figure 1C). In addition, GO and GSEA analyses were adopted to further verify the candidate genes and analyze the molecular pathways. Interestingly, *S100A9* gene expression in HCC patients was linked to “immune cell migration,” “the inflammatory response,” “hypoxia”, and “TNF-a and IL-6 signaling” (Figure 1D–1H). The above results suggested that *S100A9* gene expression was negatively associated with HCC patients’ prognosis.

### S100A9 expression is associated with a poor prognosis in HCC patients

**Figure 2:**
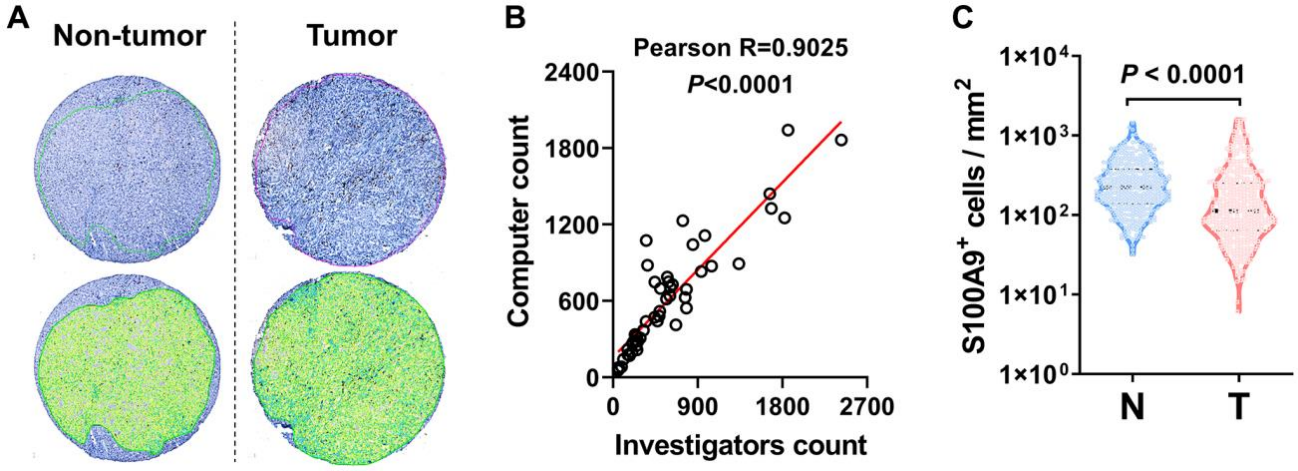
**S100A9 expression in HCC tumor.** (**A**) Representative images of IHC staining. (**B**) Scatter plot illustrating the correlation between S100A9-expressing cell counts in HCC by computer counting and investigator counting. (**C**) Quantification of S100A9^+^ cell densities in the N (nontumoral) and T (tumoral) regions (*n* = 382), and the Wilcoxon matched-pairs signed-rank test was used to analyze the nonparametric test between the paired two groups.

We performed IHC to assess the protein expression level of S100A9 in the HCC microarray chip. First, we applied automated measurements with computerized image analysis to count S100A9^+^ cells in HCC microarray assays (Figure 2A). To assess methodological consistency, we found that the number of S100A9^+^ cells determined by the automated enumeration method highly correlated with the number of positive cells detected by a pathologist (Figure 2B, Pearson R = 0.9025, *P* < 0.0001). As shown in Figure 2C, S100A9^+^ cell density was significantly higher in the nontumoral tissue than in the tumoral tissue (*P* < 0.0001).

**Figure 3:**
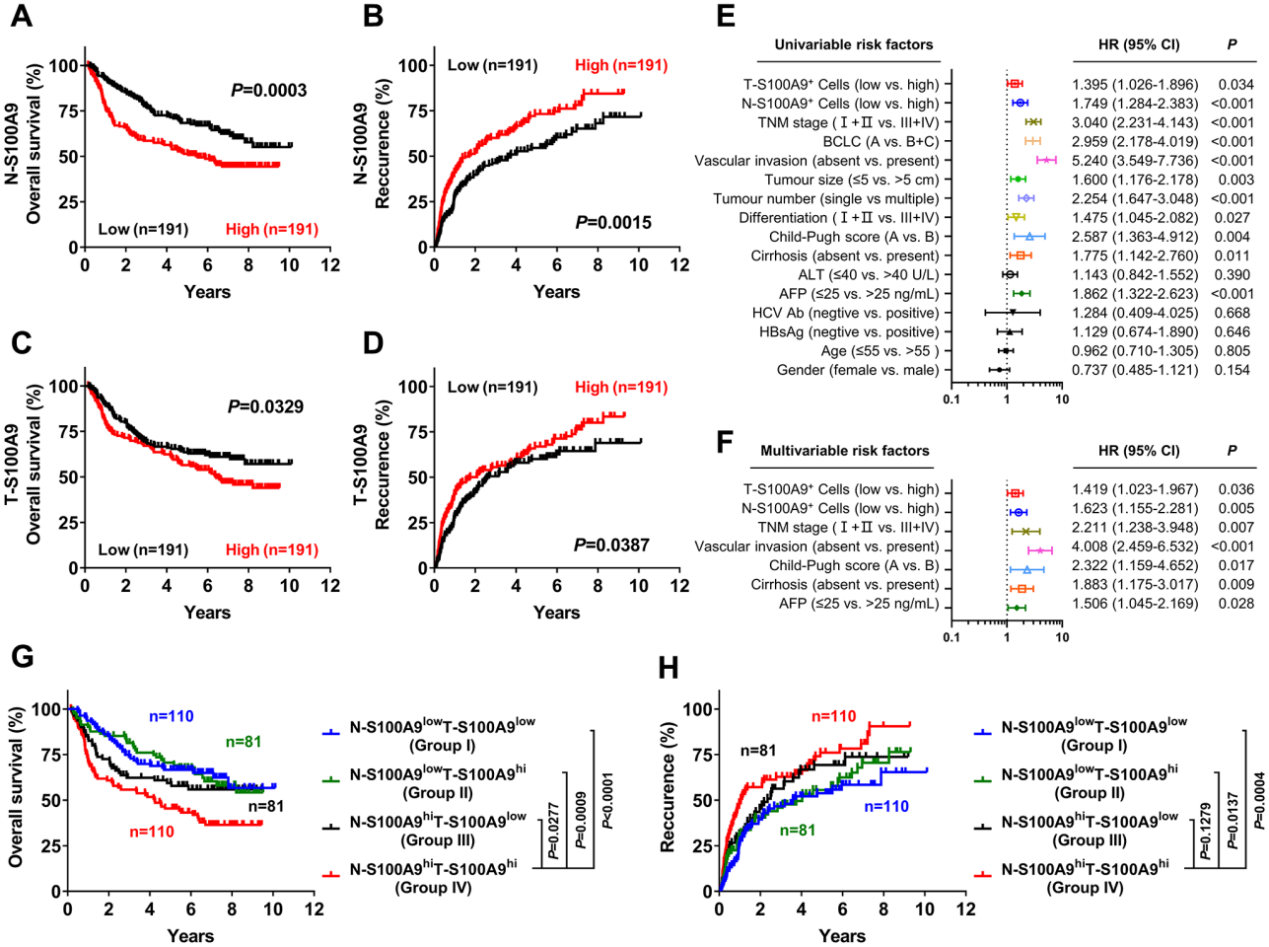
**Prognostic value of S100A9^+^ cell in HCC patients.** A high S100A9^+^ cell density in nontumor or tumor tissue was associated with poor OS (**A**, **C**) and a high recurrence rate (**B**, **D**) in HCC patients. Forest plots showing the association between S100A9 expression in tumors and clinicopathological features in HCC patients using univariate (**E**) or multivariate analysis (**F**). Patients were divided into two groups according to the median value of the S100A9^+^ cell density in nontumoral and tumoral tissues. The cumulative OS (**G**) and recurrence (**H**) times were calculated using the Kaplan-Meier method and then analyzed with the log-rank test.

We next investigated the prognostic role of S100A9 expression in HCC. A total of 382 treatment-naïve HCC patients who had long-term follow-up data were divided into two groups according to the median counts of S100A9^+^ cells in the tumoral region and nontumoral region. Kaplan-Meier analysis revealed a negative association between overall survival (OS) and the density of S100A9^+^ cells in the nontumoral region (*P* = 0.0003, Figure 3A) and tumoral region (*P* = 0.0329, Figure 3C). Patients with a high density of S100A9^+^ cells in the nontumoral region or in the tumoral region had a significantly higher recurrence rate than patients with a low density of S100A9^+^ cells (nontumor, *P* = 0.0015, Figure 3B; tumor, *P* = 0.0387, Figure 3D). According to the results of the univariate analysis (Figure 3E), S100A9^+^ cell density in the nontumoral region or in the tumoral region was associated with OS. The clinicopathologic features that were significant in univariate analysis were adopted as covariates in the multivariate analysis, which revealed that S100A9^+^ cell density in the tumor region or in the nontumoral region was a powerful independent prognostic predictor for OS (Figure 3F; tumor, HR = 1.149; *P* = 0.036; nontumor, HR = 1.623; *P* = 0.005).

We also evaluated the combined influence of S100A9^+^ cells in tumor and nontumor regions. Using the median value as a cutoff, patients were classified into four groups: I, N-S100A9^low^ T-S100A9^low^ (*n* = 110); II, N-S100A9^low^ T-S100A9^hi^ (*n* = 81); III, N-S100A9^hi^ T-S100A9^low^ (*n* = 81); and IV, N-S100A9^hi^ T-S100A9^hi^ (*n* = 110). Significant differences in OS (Figure 3G) and recurrence rate (Figure 3H) were found among the four groups. Patients in group IV exhibited the worst OS (OS rate: 45.43%) among all patients, including those in group I (5-year OS rate: 66.59%; *P* < 0.0001), group II (5-year OS rate: 70.59%; *P* = 0.0009), and group III (5-year OS rate: 59.39%; *P* = 0.0277). In addition, patients in group IV exhibited the highest recurrence rate (5-year recurrence rate: 76.07%) among all patients, including those in group I (5-year recurrence rate: 53.80%; *P* = 0.0004), group II (5-year recurrence rate: 55.53%; *P* = 0.0137), and group III (5-year recurrence rate: 69.34%; *P* = 0.1279). The above data suggest that S100A9 might represent a potential target for HCC therapy.

We also analyzed the correlation between S100A9^+^ cell density and patient clinicopathological features. Table 1 shows that the tumoral S100A9^+^ cell number did not significantly correlate with patient sex, age, HBsAg, AFP, ALT, cirrhosis, differentiation, tumor multiplicity, tumor size, Barcelona Clinic Liver Cancer (BCLC) stage or TNM stage. However, nontumoral S100A9^+^ cell density, but not tumoral S100A9^+^ cell density, was associated with sex, tumor size, BCLC stage and TNM stage.

**Table 1:** Correlation between clinicopathological parameters and the density of S100A9^+^ cells.

**Characteristics**	**No. N-S100A9^+^ (%)**			**No. T-S100A9^+^ (%)**		
	**Low (No. 191)**	**High (No. 191)**	*P* value^*^	**Low (No. 191)**	**High (No. 191)**	*P* value^*^
**Sex**						
female	30 (7.85)	14 (3.66)	**0.010**	28 (7.33)	16 (4.19)	0.054
male	161 (42.15)	177 (46.34)		163 (42.67)	175 (45.81)	
**Age, years**						
≤ 50	97 (25.39)	100 (26.18)	0.759	95 (24.87)	102 (26.70)	0.474
> 50	94 (24.61)	91 (23.82)		96 (25.13)	89 (23.30)	
**HBsAg**						
negative	23 (6.02)	16 (4.19)	0.237	20 (5.24)	19 (4.97)	0.866
positive	168 (43.98)	175 (45.81)		171 (44.76)	172 (45.03)	
**AFP, ng/mL**						
≤ 25	77 (20.16)	60 (15.71)	0.070	65 (17.02)	72 (18.85)	0.455
> 25	114 (29.84)	131 (34.29)		126 (32.98)	119 (31.15)	
**ALT, U/L**						
≤ 40	117 (30.63)	102 (26.70)	0.121	106 (27.75)	113 (29.58)	0.469
> 40	74 (19.37)	89 (23.30)		85 (22.25)	78 (20.42)	
**Cirrhosis^†^**						
absent	39 (10.43)	42 (11.23)	0.706	45 (12.03)	36 (9.63)	0.236
present	148 (39.57)	145 (38.77)		141 (37.70)	152 (40.64)	
**Differentiation^†^**						
I + II	154 (40.63)	141 (37.20)	0.131	151 (39.84)	144 (37.99)	0.248
III + IV	36 (9.50)	48 (12.66)		37 (9.76)	47 (12.40)	
**Tumor multiplicity**						
single	145 (37.96)	135 (35.34)	0.247	146 (38.22)	134 (35.08)	0.165
multiple	46 (12.04)	56 (14.66)		45 (11.78)	57 (14.92)	
**Tumor size, cm**						
≤ 5	117 (30.63)	71 (18.59)	**< 0.001**	93 (24.35)	95 (24.87)	0.838
> 5	74 (19.37)	120 (31.41)		98 (25.65)	96 (25.13)	
**BCLC**						
A	137 (35.86)	108 (28.27)	**0.002**	130 (34.03)	115 (30.10)	0.110
B + C	54 (14.14)	83 (21.73)		61 (15.97)	76 (19.90)	
**TNM stage**						
I + II	153 (40.05)	122 (31.94)	**< 0.001**	142 (37.17)	133 (34.82)	0.305
III + IV	38 (9.95)	69 (18.06)		49 (12.83)	58 (15.18)	

^†^Data were missing in these variables for some patients.

^*^χ^2^ test. Significant *P* values (< 0.05) are shown in bold.

### S100A9 expression correlates with myeloid cell infiltration in the tumor microenvironment

**Figure 4:**
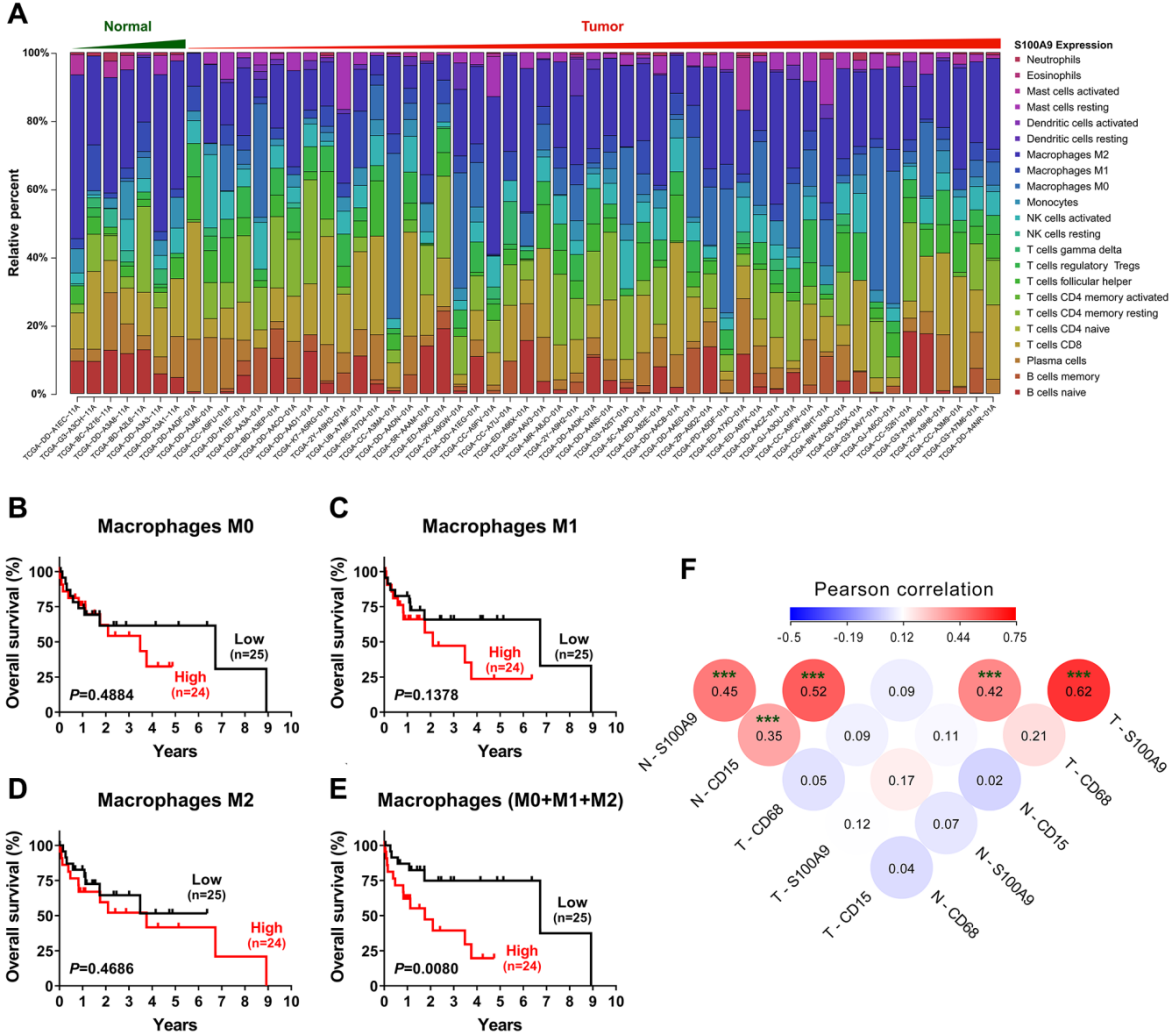
**Tumor-infiltrating immune cells associated with S100A9 expression in HCC.** (**A**) Differential infiltration of immune cells based on *S100A9* expression in groups from the TCGA-LIHC cohort. The proportions of 22 immune infiltrates in tumor (*n* = 49) and nontumor tissue (*n* = 7) were estimated using the CIBERSORT algorithm. (**B**–**E**) Prognostic value of M0, M1, M2 and total (M0 + M1 + M2) macrophage densities in HCC (*n* = 49). (**F**) The densities of S100A9^+^ cells and CD15^+^ neutrophils and CD68^+^ macrophages were analyzed using Pearson correlation analysis (*n* = 382). ^***^P < 0.001.

To investigate the relationship between S100A9 expression and the immune system, we adopted the CIBERSORT algorithm [18] to evaluate the association between *S100A9* gene expression and tumor immune cell infiltration using the TCGA-LIHC data set. Only 7 nontumoral and 49 tumoral samples with a CIBERSORT *P*-value < 0.05 were retained for further study. The abundances of twenty-two immune cell types in each HCC sample are presented in the boxplot in Figure 4A. The results indicated that the infiltration of immune cells was not significantly related to the expression of the *S100A9* gene (data not shown). However, in terms of the component of cells, macrophages (M0 + M1 + M2 Mφ) were the main cell populations in the microenvironment of HCC tumor tissue. Hence, we investigated the prognostic significance of Mφs in 49 patients in the TCGA-LIHC dataset. As shown in Figure 4B–4E, the M0, M1 and M2 Mφ densities did not correlate with the survival of HCC patients. However, patients with a high density of Mφs had significantly shorter OS than patients with a low Mφ density. Moreover, the association among the density of S100A9^+^ cells, CD15^+^ neutrophils and CD68^+^ Mφ infiltration in our tissue microarray was analyzed (Figure 4F). The density of S100A9^+^ cells was positively associated with the infiltration of CD15^+^ cells and CD68^+^ cells in both the nontumoral and tumoral regions.

### Cellular source of S100A9^+^ cells

**Figure 5:**
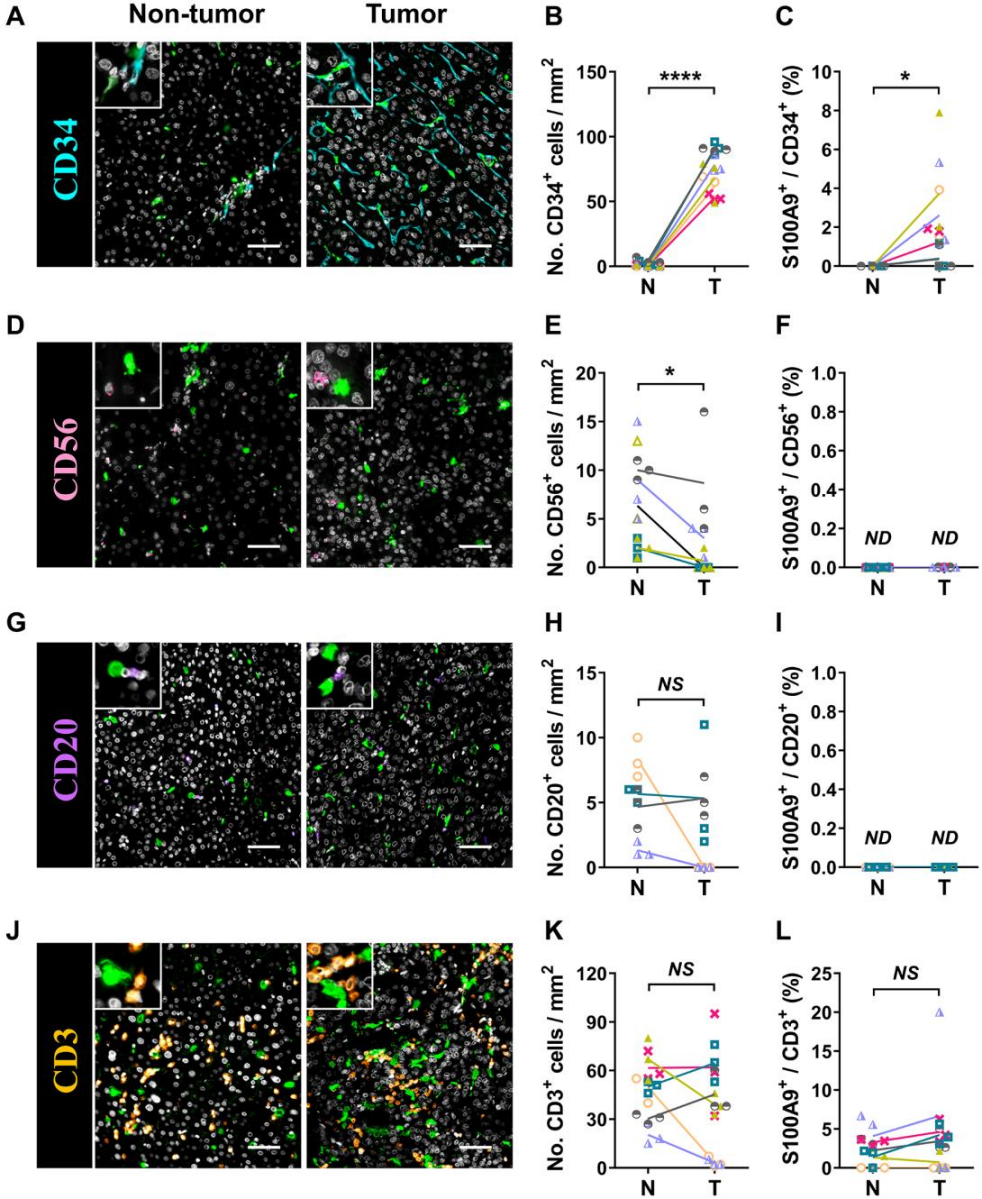
**Characterization of patient-derived S100A9^+^ cells.** Multiple immunofluorescence staining shows DAPI (gray), S100A9 (green), CD34 (blue, **A**), CD56 (pink, **D**), CD20 (purple, **G**), and CD3 (orange, **J**) expression and coexpression (double-positive cells) in HCC tissue. Quantification of CD34 (**B**), CD56 (**E**), CD20 (**H**), and CD3 (**K**) cell densities in the T and N regions. (**C**) The percentages of S100A9^+^CD34^+^ cells among the total CD34^+^ cells in the N and T regions. (**F**) The percentages of S100A9^+^CD56^+^ cells among the total CD56^+^ cells in the N and T regions. (**I**) The percentages of S100A9^+^CD20^+^ cells among the total CD20^+^ cells in the N and T regions. (**L**) The percentages of S100A9^+^CD3^+^ cells among total CD3^+^ cells in the N and T regions (*n* = 4 - 7). Scale bar = 50 μm. The results are the means ± SEM (bars); *NS*, no significance; *ND*, not detected.

**Figure 6:**
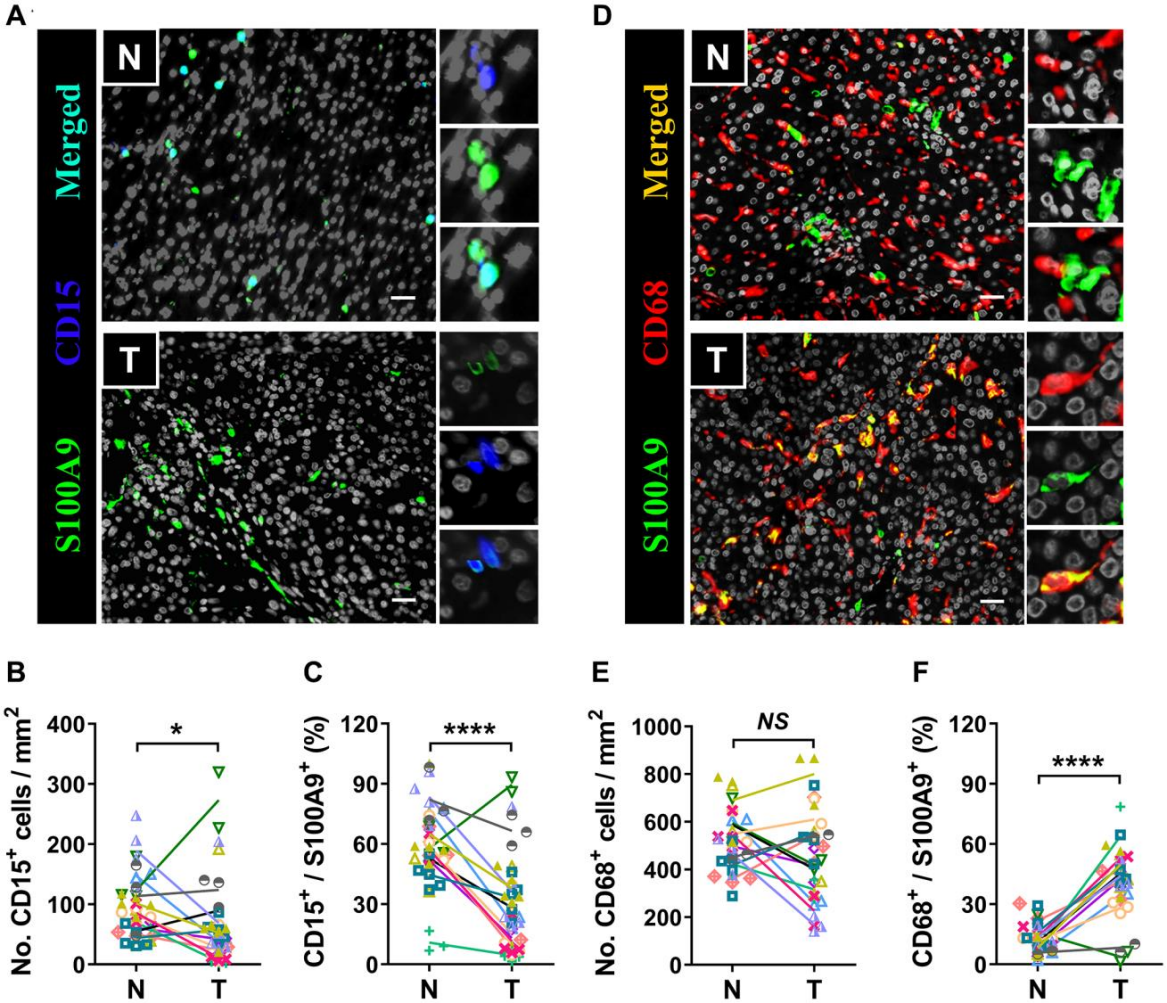
**Myeloid cells are the major source of S100A9.** Multiple immunofluorescence staining shows DAPI (gray), S100A9 (green), CD15 (blue, **A**), and CD68 (red, **D**) expression and coexpression (double-positive cells) in the N and T regions. Quantification of CD15^+^ (**B**) and CD68^+^ (**E**) cell densities in the T and N regions (*n* = 12). (**C**) The percentages of S100A9^+^CD15^+^ cells among the total S100A9^+^ cells in the N and T regions. (**F**) The percentages of S100A9^+^CD68^+^ cells among the total S100A9^+^ cells in the N and T regions. (*n* = 12). Scale bar = 25 μm. N, nontumor, T, tumor. ^*^P < 0.05, ^****^P < 0.0001, *NS*, no significance.

S100A9 is expressed in a heterogeneous population of cells [7, 8]. Multicolor immunofluorescence staining was performed to characterize the cellular source of S100A9^+^ cells in HCC. S100A9 was expressed at low levels in CD34^+^ endothelial cells and CD3^+^ T cells and was not detected in CD56^+^ NK cells or CD20^+^ B cells (Figure 5). As expected, S100A9 protein was mainly expressed in myeloid cells. We found that neutrophils predominantly infiltrated the nontumoral area rather than the tumoral area, and CD15^+^ cells were the main cell population that expressed S100A9 protein (58.19 ± 20.80%) in nontumor areas, but this proportion was significantly reduced in tumor areas (29.01 ± 24.20%) (Figure 6A–6C). In addition, 39.69 ± 17.30% of S100A9^+^ cells in the tumoral region were CD68^+^ cells, but only 12.23% ± 7.17% of S100A9^+^ cells in the nontumoral region were CD68^+^ cells (Figure 6D–6F). Collectively, our findings indicated that the primary S100A9-expressing cells in adjacent nontumoral tissue are CD15^+^ neutrophils, and both CD68^+^ Mφs and CD15^+^ neutrophils highly express S100A9 in HCC tumor tissues.

## DISCUSSION

S100 proteins are involved in a variety of biological processes and expressed in various cell types in tissues. We delineated the distribution and prognostic value of S100A9^+^ cells in HCC tissue. A high S100A9^+^ cell density in either tumoral or nontumoral tissue was found to be a predictor of unfavorable prognosis and could serve as an independent risk factor in patients with HCC. Furthermore, we demonstrated that Mφs and neutrophils accounted for most S100A9^+^ cells in HCC tumoral tissue and that neutrophils were the dominant S100A9^+^ cells distributed in nontumoral tissue.

S100A9, a member of the S100 family, is expressed in granulocytes, monocytes, macrophages, MDSCs and tumor cells in various cancers [7, 8, 17, 19, 20]. In this study, we found that S100A9 was predominately located in infiltrating Mφs and neutrophils in HCC tumoral tissue. In nontumoral tissue, neutrophils were the main source of S100A9^+^ cells. Few HCC cells themselves express S100A9. These results were consistent with those of previous studies demonstrating high S100A9 expression in infiltrating immune cells in various cancer types, including colorectal cancer and pancreatic cancer [9, 11]. In other cancer types, such as lung cancer [21], prostate cancer [22], nasopharyngeal carcinoma [23], and breast cancer [24], S100A9 is expressed mainly by neoplastic tumor cells themselves. Arai et al. found that S100A9 is expressed in malignant hepatocytes and that high S100A9 expression is related to poorly differentiated carcinomas [25]. However, we found few S100A9-positive HCC cells, even in poorly differentiated HCC. The inconsistent results are probably due to differences in the stage, number, and size of tumors; the sample size or the antibodies used to detect S100A9 expression. Overall, high S100A9 expression on Mφs and neutrophils in HCC tissues may indicate that S100A9 plays a crucial role in HCC development.

The relationship between S100A9 expression in HCC and disease progression remains unclear. Here, we observed that high tumoral S100A9 expression at both the RNA (TCGA-LIHC data set analysis) and protein (IHC detection) levels was associated with a poor prognosis. Furthermore, multivariate analyses revealed that S100A9^+^ cell number was an independent and significant prognostic factor in HCC. Consistent with our results, previous studies showed that S100A9^+^ cell density was associated with a poor prognosis in patients with other cancers, such as clear cell renal cell carcinoma [26], invasive ductal carcinoma of the breast [24], and non-small cell lung cancer [21].

S100A9 plays an important role in malignant development and cancer progression by triggering protumor immune responses. Previous studies have shown that S100A9 acts as a chemotactic molecule to recruit inflammatory cells or immunocytes, such as MDSCs and neutrophils [27, 28], to the tumor microenvironment, resulting in a proinflammatory microenvironment that promotes tumor progression [29, 30]. We found that high S100A9 expression in HCC patients was linked to macrophage chemotaxis, mast cell activation, mononuclear cell migration, and eosinophil migration. These immune cells in the tumor microenvironment can produce vast amounts of cytokines, growth factors and chemokines as well as reactive oxygen species (ROS) and nitric oxide (NO), which stimulate proliferation, promote stemness, prevent apoptosis, induce morphogenesis, mediate DNA damage in epithelial cells [31–33], and promote the survival and migration of cancer cells [34–37]. Furthermore, S100A9 also acts as a novel NF-κB target gene in HCC cells [36, 38]. S100A9 can efficiently activate immune cells in the microenvironment to secrete TNF-α and maintain the protumor phenotype [39, 40]. Consequently, S100A9 might control tumor progression by acting either directly on tumor cells or indirectly on the tumor microenvironment.

Interestingly, S100A9 staining of the nontumor tissue of HCC patients provided prognostic information. Current evidence has demonstrated that tumor-adjacent, morphologically normal tissue is not completely normal [41–43]. S100A9 was expressed in nontumor tissue, suggesting the early involvement of the proteins in HCC.

However, this study has certain limitations. The underlying molecular mechanisms regulating distinct cell sources of S100A9^+^ cells in nontumoral and tumoral regions are still largely unclear, further studies are needed. Nevertheless, our current study identified that high S100A9 expression was associated with poor OS and a high recurrence risk in patients with HCC. Consequently, S100A9 expression could be considered a significant prognostic marker in patients with HCC.

## MATERIALS AND METHODS

### Patients and tissue specimens

This study was approved by the Ethics Committee of Sun Yat-sen University Cancer Center (SYSUCC). A total of 382 patients with pathologically confirmed HCC who underwent surgery at the SYSUCC from June 21, 2006 to September 17, 2010 were included in this study. The patients provided informed consent for participation in the present study. The patients did not receive any immunotherapy or neoadjuvant therapy before the operation. The clinical data of the patients were extracted from their electronic medical records and are listed in Table 1.

The clinical stage of tumors was determined according to the tumor-node-metastasis (TNM) classification system of the American Joint Committee on Cancer (AJCC, 2018–01–01, 8th edition). The follow-up period ended in July 2014, and the median survival time was 63 months (range, 1 – 121 months). Recurrence was diagnosed pathologically via surgical biopsy and/or radiologically via computed tomography or positron emission tomography. OS was defined as the length of time between surgery and death or the last follow-up examination.

### Analysis of data from The Cancer Genome Atlas (TCGA)

Data on the expression levels of the *S100A9* gene in human HCC were obtained from TCGA up to April 17, 2020. Samples included only those that were annotated as “untreated, primary HCC”. A total of 365 HCC tissues and 50 normal tissues were selected based on the official TCGA**-**liver hepatocellular carcinoma (LIHC) dataset. *S100A9* gene expression (as transcripts per kilobase million (TPM) values) and corresponding clinical and pathological data of these samples were downloaded and processed as previously described. The gene expression TPM data of *S100A9* are shown in Supplementary Table 1.

### Gene ontology (GO) analysis and gene set enrichment analysis (GSEA)

According to the expression level of S100A9 in the tumor tissues of 365 patients in the TCGA-LIHC database, the patients were divided into high and low groups (low, *n* = 183; high, *n* = 182).

Under the condition of *P* < 0.05, a total of 3493 genes were highly expressed in the *S100A9*^high^ group (the fold change of high group/low group > 2), and 399 genes were highly expressed in the *S100A9*^low^ group (the fold change of high group/low group < 0.5). These differentially expressed genes are shown in Supplementary Table 2. The top 30 GO terms (Supplementary Table 3) of the 3493 genes highly expressed in the *S100A9*^high^ group were analyzed using GO enrichment tools (http://enrich.shbio.com/index/ga.asp).

All transcriptome expression results of *S100A9*^low^ and 182 *S100A9*^high^ patients were subjected to 50 hallmark gene analyses of sets by using GSEA software (version 4.0.2, http://software.broadinstitute.org/gsea/index.jsp). The detailed results are shown in Supplementary Table 4.

### Immunohistochemistry (IHC) and immunofluorescence

A tissue microarray (TMA) was constructed, and the slides were incubated with a rabbit monoclonal primary antibody against S100A9 (34425, 1:2000, Cell Signaling Technology, USA) at 4°C overnight. The sections were stained using an Envision system (Dako; Carpinteria, CA, USA) as previously described [44].

Double immunofluorescent staining was performed as previously described [45]. Briefly, formalin-fixed paraffin-embedded (FFPE) sections were incubated at 4°C overnight with the following primary antibodies: mouse anti-human CD15 (1:200; ZSBio, China); CD68 (1:200; Dako Cytomatin, USA); CD3 (1:200; ZSBio, China); rabbit anti-human CD20 (1:200; Abcam, UK); CD34 (1:200; ZSBio, China); and CD56 (NCAM1) (1:1000; Sino Biological Inc, China). The sections were then incubated for 30 min at 37°C with a mixture of primary-antibody matched fluorescently labeled secondary antibodies (1:500; Invitrogen; CA, USA).

### Image analysis

Immunohistochemically and fluorescently stained sections were scanned at a magnification of ×20 using the Aperio Digital Pathology Scanner (Leica Biosystems Inc., Germany) to capture a digital whole slide image. Digital image analysis for quantification of the staining for S100A9, CD34, CD56, CD20, CD3, CD15 and CD68 was measured using Aperio ScanScope AT Turbo, eSlide Manager and ImageScope software (V12.3.3.7014; Leica Biosystems, Vista, CA, USA) in accordance with the manufacturer’s recommendations. The 3 most representative high-power fields were captured for each tumor and nontumor region in all specimens. All measurements were performed by the same researcher, who was blinded to the histologic and patient survival data, to prevent interoperator variability. Moreover, the protein expression level of S100A9 (cells/mm^2^) in the immunohistochemical staining of the tissue microarray is shown in Supplementary Table 5.

### Statistical analyses

All statistical analyses were performed using SPSS version 25.0 (SPSS Inc., Chicago, IL, USA) and GraphPad Prism (version 8). The Kolmogorov-Smirnov test and Shapiro-Wilk test were used to analyze normality. The significance of differences between groups was determined by the Mann-Whitney test and Wilcoxon matched-pairs signed-rank test. Pearson’s correlation was used to evaluate the correlation between investigator count and computer count, and to evaluate the correlation of variables with immune cell infiltration. Survival curves were assessed by Kaplan-Meier analysis with the log-rank test. The Cox proportional hazards model was used to identify prognostic factors through univariate and multivariate analyses. The statistical significance of differences between groups was determined using the two-tailed Student’s *t*-test, where differences with *P* < 0.05 were considered significant.

### Ethics approval and consent to participate

All patients’ samples were anonymously coded in accordance with local ethical guidelines (as stipulated by the Declaration of Helsinki), and written informed consent was provided. The Review Board of Sun Yat-sen University Cancer Center approved the study protocol.

## Supplementary Materials

Supplementary Table 1

Supplementary Table 2

Supplementary Table 3

Supplementary Table 4

Supplementary Table 5
